# Epilepsy

**Published:** 1863-08

**Authors:** J. S. Ramskill


Epilepsy—*Belladonna.*—At first the dose should be very small, and gradually augmented until the pupil shows signs of its action, and the patient complains of both alteration in sight and dryness of the throat; the dose must then be diminished until these effects are so far lessened as to cease causing absolute discomfort. The other toxic effects of belladonna are wholly uncalled for. Immediate and palpable beneficial results must not be looked for. At first the number of fits may even increase, but after the drug has been taken six or eight weeks, if any amelioration occur, it will be decided and progressive. At first the drug should be given in an eighth of a grain dose, three times, or only twice daily, for a week; then a quarter of a grain for fourteen days; a third for the next fourteen days, at which time its physiological action will in most cases be manifest. It is well to halt at this dose, for two or three months, slightly increasing the dose if the patient shows diminished susceptibility to its influence. To those who wish to use a preparation of uniform strength. the salts of atropia are easily procurable. The hundred-and-twentieth of a grain of valerianate of atropia may be given as a commencing dose. Trousseau gives a centigramme of the extract, and an equal quantity of the powder of belladonna for the first month, in the evening of each day; during the second month he gives two such pills at the same time; and during the third month, three pills If, at the end of six or nine months, the frequency of the fits is decreased, he increases the dose. He asserts that, of 120 patients, he has cured 20, (Dr. J. S, Ramskill.)—*Id.*

